# Systematic analysis reveals tumor-enhancing and -suppressing microRNAs in *Drosophila* epithelial tumors

**DOI:** 10.18632/oncotarget.22226

**Published:** 2017-11-01

**Authors:** Zhiqiang Shu, Yi-Chun Huang, William H. Palmer, Yoichiro Tamori, Gengqiang Xie, Hui Wang, Nan Liu, Wu-Min Deng

**Affiliations:** ^1^ Department of Biological Science, Florida State University, Tallahassee, Florida, USA; ^2^ Interdisciplinary Research Center on Biology and Chemistry, Shanghai Institute of Organic Chemistry, Chinese Academy of Sciences, Shanghai, China; ^3^ Current/Present address: Institute of Evolutionary Biology, University of Edinburgh, Edinburgh, UK; ^4^ Current/Present address: Structural Biology Center, National Institute of Genetics and Department of Genetics, School of Life Science, SOKENDAI (The Graduate University for Advanced Studies), Mishima, Japan

**Keywords:** miRNA, RNA-Seq, lgl, nTSGs, tumorigenesis

## Abstract

Despite their emergence as an important class of noncoding RNAs involved in cancer cell transformation, invasion, and migration, the precise role of microRNAs (miRNAs) in tumorigenesis remains elusive. To gain insights into how miRNAs contribute to primary tumor formation, we conducted an RNA sequencing (RNA-Seq) analysis of *Drosophila* wing disc epithelial tumors induced by knockdown of a neoplastic tumor-suppressor gene (nTSG) *lethal giant larvae* (*lgl*), combined with overexpression of an active form of oncogene *Ras* (*Ras*^*V12*^), and identified 51 mature miRNAs that changed significantly in tumorous discs. Followed by *in vivo* tumor enhancer and suppressor screens in sensitized genetic backgrounds, we identified 10 tumor-enhancing (TE) miRNAs and 11 tumor-suppressing (TS) miRNAs that contributed to the nTSG defect-induced tumorigenesis. Among these, four TE and three TS miRNAs have human homologs. From this study, we also identified 29 miRNAs that individually had no obvious role in enhancing or alleviating tumorigenesis despite their changed expression levels in nTSG tumors. This systematic analysis, which includes both RNA-Seq and *in vivo* functional studies, helps to categorize miRNAs into different groups based on their expression profile and functional relevance in epithelial tumorigenesis, whereas the evolutionarily conserved TE and TS miRNAs provide potential therapeutic targets for epithelial tumor treatment.

## INTRODUCTION

microRNAs (miRNAs), approximately 22 nucleotides (nt) in length, are a group of endogenous, non-protein-coding, small RNAs [1]. Since their discovery in *Caenorhabditis elegans* in 1990s, an increasing number of miRNAs have been identified in multicellular eukaryotes [1–3]. Canonical miRNA production starts with long primary RNA transcripts (pri-miRNAs), which are cleaved by a ribonuclease complex comprising two proteins, drosha and pasha, to create ∼60-70-nt precursor miRNAs (pre-miRNAs) [4]. These pre-miRNAs are then transported to the cytoplasm where they are cut into small ∼22 nt RNAs by a second ribonuclease, Dicer-1 (Supplementary Figure 1A) [5]. For miRNAs to function, they are incorporated into the RNA-induced silencing complex (RISC), and the miRNA/RISC complex then binds to partially complementary sequences of target mRNAs for degradation or translational repression (Supplementary Figure 1B) [6, 7].

miRNAs are pivotal post-transcriptional gene expression regulators, playing indispensable roles in regulating various biological functions and processes, including cell identity, metabolism, and reproduction [1]. For example, *miR-125* and *let-7*, the *Drosophila* homologs of *lin-4* and *let-7* in *C. elegans*, play a role in temporal regulation of metamorphic processes [8]. A well-conserved miRNA *miR-7* plays key roles in multiple gene networks to maintain eye homeostasis against temperature perturbation and ovarian follicle cells development [9, 10]. More recently, miRNAs have emerged as a powerful player in tumorigenesis. Studies have shown that miRNAs are implicated in various types of human cancer, including lung, breast, brain, liver, colon cancer, and leukemia [11]. For example, the *mir-17-92* cluster was shown to be overexpressed in human lung cancers [12], suggesting its oncogenic role. In contrast, *let-7* was significantly reduced in lung cancer [13], suggesting that it may act as a tumor suppressor. In *Drosophila*, a handful of miRNAs have been mechanistically studied about their roles in growth regulation. For example, *bantam* is implicated in hyperplastic overgrowth by targeting pro-apoptotic gene *hid* [14]. *miR-8* targets Notch ligand Serrate, and therefore was identified as a potent inhibitor of Notch-induced overgrowth and tumor metastasis [15]. Although the relationship between cancer and miRNAs is well documented, a holistic picture of how miRNAs contribute to tumorigenesis is lacking.

*Drosophila*, the fruit fly, has been employed to model various forms of human cancers, and thus provided many illuminating discoveries in the basic research and therapeutic spaces [16]. Research on a group of conserved epithelial cell polarity genes, *lethal giant larvae* (*lgl*), *discs large* (*dlg*), and *scribble* (*scrib*) revealed their indispensable roles in maintaining apical-basal cell polarity and epithelial tissue organization [17, 18]. In *Drosophila, dlg* and *scrib* both encode scaffolding proteins, which are found throughout the basolateral domain of the epithelia and support the septate junction [19]. Though it lacks similar scaffolding structure, *lgl* also acts on the basolateral side of epithelial cells, antagonizing the activity of apical proteins Bazooka/Par3 and aPKC [20]. Therefore, depletion of any of these genes results in polarity disruption and induces malignant epithelial tumors [18, 21]. Similarly, loss of the homologs of these genes in mammals displays development and progression of malignant tumors [22].

Here, we modeled malignant epithelial tumors in the *Drosophila* wing imaginal disc by knocking down *lgl* and expressing an oncogenic *Ras*. We conducted RNA-Seq on the tumor tissues and normal tissues to carry out a comprehensive survey of miRNAs and identified 51 of them that were differentially expressed. Using tumor enhancer and suppressor screens, we identified two groups of miRNAs that actively contribute to this tumorigenesis. Ten upregulated miRNAs that could enhance *lgl* knockdown-induced tumorigenesis are named “tumor-enhancing (TE) miRNAs,” due to their active role in collaborating with nTSGs in promoting epithelial tumorigenesis. Similarly, we name 11 downregulated miRNAs that inhibited nTSG defect-induced tumorigenesis “tumor-suppressing (TS) miRNAs.” We show that some of these *Drosophila* tumor-implicated miRNAs are conserved in humans and have been reported to be involved in human cancers. Our studies systematically analyze miRNAs in epithelial tumors and elucidate their causative roles in tumorigenesis.

## RESULTS

### *lgl-knockdown/Ras*^*V12*^-induced epithelial tumors in *Drosophila* wing imaginal discs

**Figure 1:**
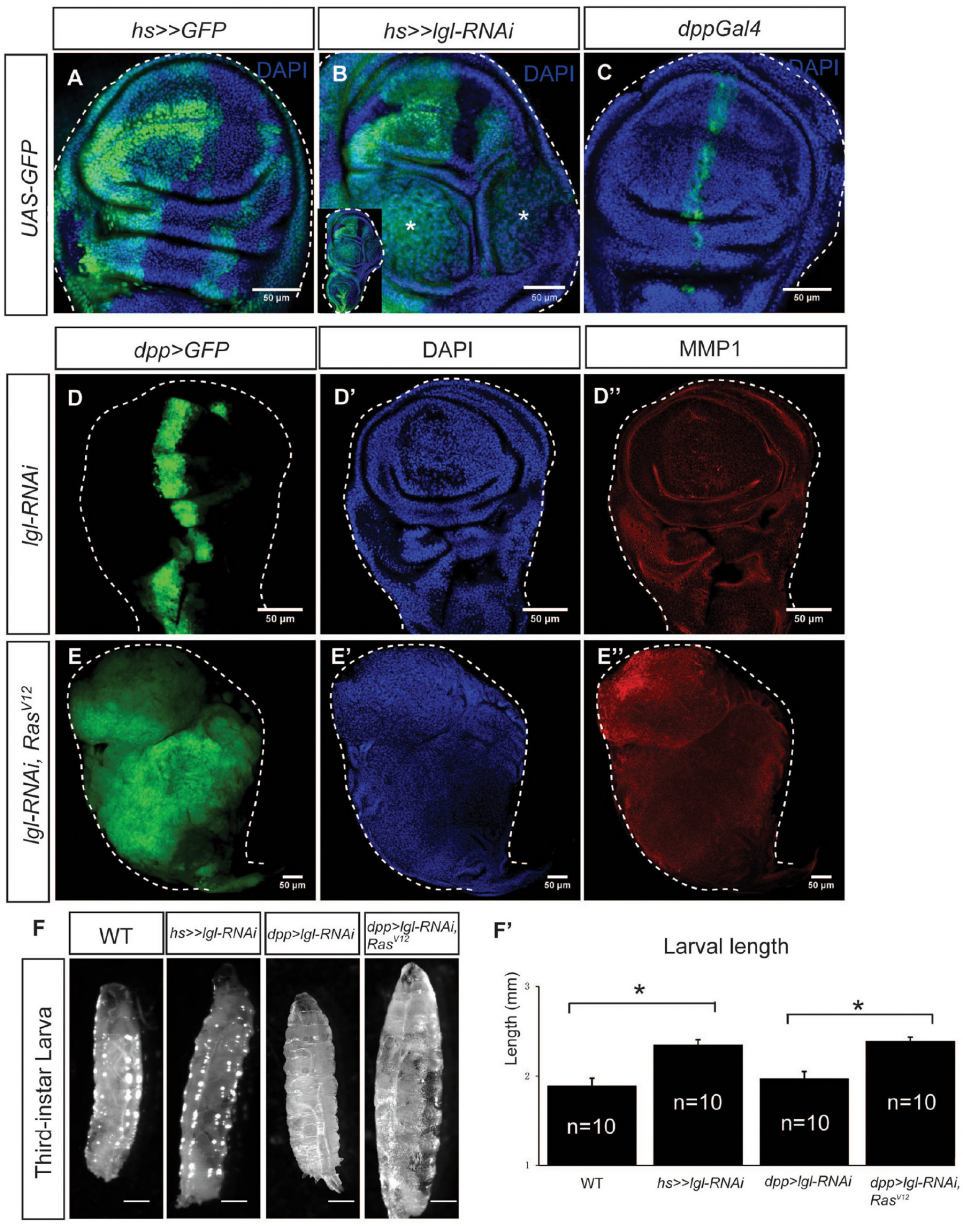
*lgl-knockdown/Ras*^*V12*^-induced epithelial tumors in *Drosophila* wing imaginal discs. **(A)** The flip-out Gal4 system expressed the marker protein GFP in the wing imaginal disc, forming random cell clusters. **(B)** Tumors were generated by *hsFlp>>lgl-RNAi*. The enlarged whole disc is shown in the inset. Stars indicate the tumors. **(C)** GFP was expressed by *dpp-Gal4* on the anterior/posterior boundary of the wing imaginal disc. **(D-D’’)** *dpp-Gal4* drove *lgl-RNAi* expression in the wing disc, marked by GFP (D) The wing disc showed normal tissue organization (D’) and no MMP1 upregulation (D’’). **(E-E’’)** *dpp-Gal4* drove *lgl-RNAi* and *Ras*^*V12*^ co-expression in the wing disc, marked by GFP (E), and the disc showed overgrowth (E’) and MMP1 upregulation (E’’). Scale=50 um. **(F-F’’)** The giant larva phenotype was observed when tumors were generated in the wing disc. Scale= 2.5mm. The size increases were significant (F’), n=10, p-value <0.05.

The epithelial sheet of the *Drosophila* wing imaginal disc is composed of columnar epithelial cells, forming the pseudostratified monolayer, where cells maintain apical-basal polarity [21]. Knockdown of an nTSG *lgl* by using the Flip-out UAS/Gal4 technique induced cell overproliferation and epithelial tumorigenesis (Figure 1A–1B); however, the tumors were only generated in the tumor “hotspots,” where a tissue environment favorable for tumor growth is locally formed [21]. When we used *dpp-Gal4*, which drives gene expression on the anterior-posterior boundary in the wing disc (Figure 1C), to express *lgl-RNAi*, the wing disc maintained normal morphology, and matrix metalloproteinase 1 (MMP1), a marker for tumorigenesis and potential metastasis, was not obviously upregulated (Figure 1D–1D’’). This is likely due to cell competition-induced apoptosis on the boundary between *lgl*-knockdown cells and wildtype cells (Figure 1C) [21]. It has been reported that more prevalent, aggressive epithelial tumors can be generated by combining the depletion of a tumor suppressor, such as *lgl*, with the activation of an oncogene, such as *Ras* [23, 24, 25]. Indeed, we knocked down *lgl* by *decapentaplegic* (*dpp)-Gal4 driven lgl-RNAi*, combined with activation of Ras by expressing *Ras*^*V12*^ in the wing disc, and found that the wing discs displayed drastic overgrowth and the entire disc became tumorous (Figure 1E–1E’’). Moreover, five days after the *lgl-RNAi* induction, the larvae showed the giant larva phenotype and the increases in body size were significant (Figure 1F–1F’, n=10, p-value < 0.05). In the following studies, we used this easily recognizable giant larva phenotype as an indication of tumorigenesis [18]. To test whether the tumorous discs had the potential to continuously overgrow, we transplanted portions of tumorous wing discs containing *dpp>lgl-RNAi* and *Ras*^*V12*^ into the abdomen of adult female fly flies and examined the implanted tissue growth in the host animals. We found that, at 29°C 12 days after transplantation, host animals had a large tumorous tissue derived from the transplant in the abdomen (Supplementary Figure 2A–2B’), and the tumors had invaded and metastasized into the ovaries (Supplementary Figure 2C–2C’), These results confirm the ability of cells with a *dpp>lgl-RNAi/Ras*^*V12*^ background to dramatically overgrow and metastasize nearby tissues. As this genetic combination (*dpp>lgl-RNAi/Ras*^*V12*^) gives a severe tumorigenesis phenotype, we used it to generate tumor tissues for subsequent systematic analysis.

### Small RNA-Seq results show miRNA differential expression

The use of next-generation sequencing technology for RNA sequencing (RNA-Seq) has facilitated the discovery of novel miRNAs. As of the most recent release (release 21) from miRbase.org, there are 256 precursors and 466 mature miRNAs recognized in *Drosophila melanogaster* [26], To assess the miRNA expression globally in nTSG defect-induced epithelial tumors in the wing disc, we leveraged the small RNA-Seq technique to determine miRNA expression levels in epithelial tumors in the *Drosophila* wing disc. The small RNA-Seq analysis was conducted as shown in Supplementary Figure 3A and described in Materials and Methods. We removed low-quality reads and selected reads between 18- to 35- nt inlength, and then mapped these sequences to that of the 466 mature miRNAs in *Drosophila melanogaster* [26]. On average across three samples, per 10 million raw reads, 19,001,658 reads were found in the wildtype, 8,994,225 of which were mapped to the known miRNAs; and 11,671,543 reads were identified in the tumorous wing disc, 6,053,895 of which were mapped to the known miRNAs (Supplementary Table 1). The miRNAs in the normal and tumor tissues showed a comparable range of length distribution (Supplementary Figure 3B), suggesting normal processing and trimming on the miRNAs.

**Figure 2:**
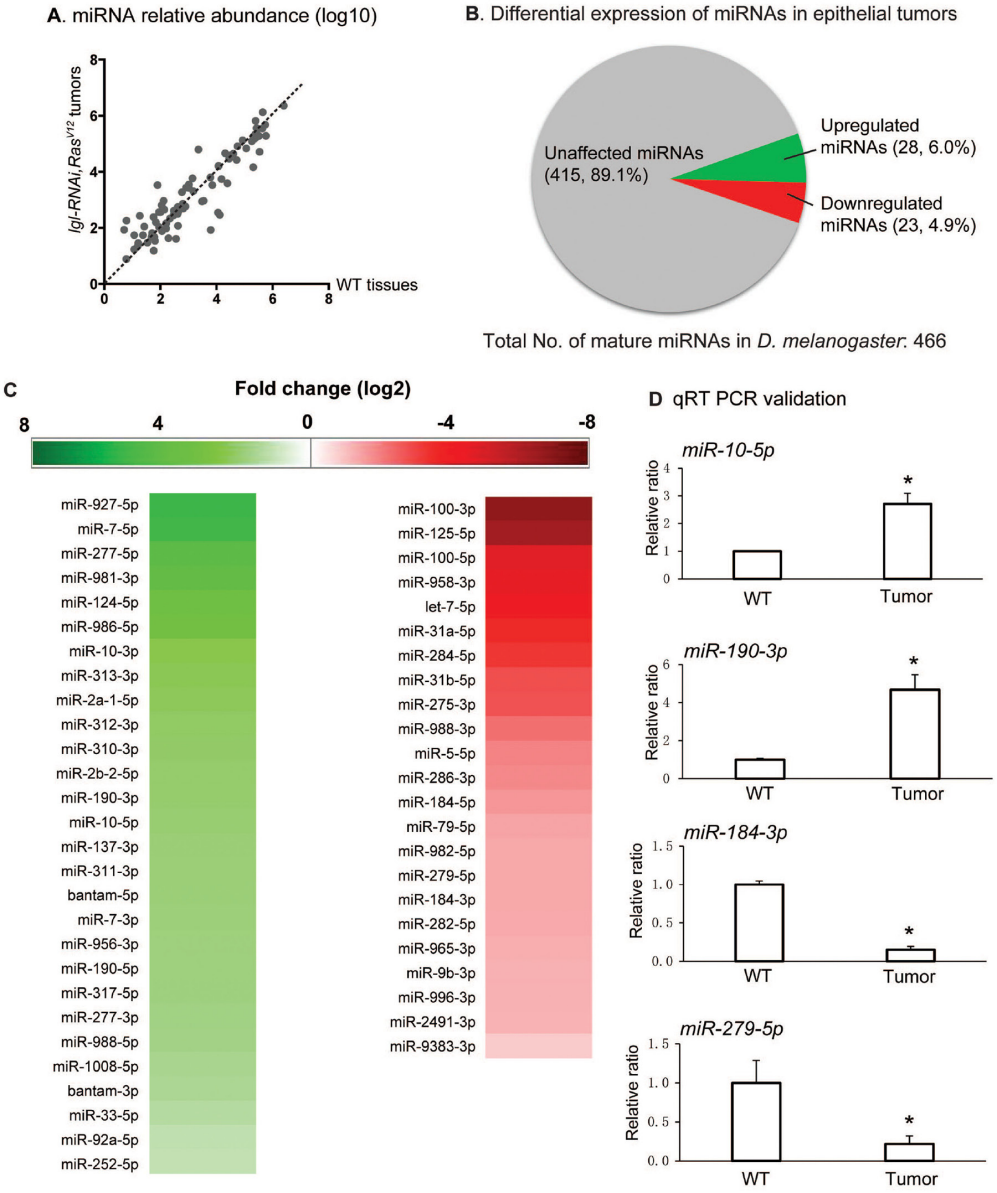
Small RNA-seq results show miRNA differential expression. **(A)** Scatter plot for miRNA relative abundance, showing a comparison of the relative abundance of the 466 *D. melanogaster* miRNAs in *lgl-RNAi, Ras*^*V12*^ tumor tissues and wildtype (WT) tissues. The miRNA read counts were calculated by miRDeep2, normalized with DESeq and transformed by log 10. **(B)** Pie chart showing differential expression of miRNA in epithelial tumors. miRNAs with no less than 1 as the absolute value of log2 ratio and had at least 50 reads per 10 million raw reads in either samples were selected as upregulated or downregulated miRNAs. 28 miRNAs were identified as upregulated, comprising 6.0% of total miRNAs, and 23 were identified as downregulated miRNAs, comprising 4.9% of the total miRNAs. **(C)** Upregulated and downregulated miRNAs in *lgl-RNAi, Ras*^*V12*^ tumor tissues are listed. The shades of the color indicate levels of the change. **(D)** Quantitative RT-PCR was conducted on miR-10, miR-190, miR-184, and miR-279 to confirm their miRNA changes. Stars indicate the significant differences. Sample size n=3, p-value < 0.05.

We then compared the relative expression level of individual miRNAs in tumors against those in wildtype wing disc tissues. Interestingly, the miRNAs demonstrated substantial difference in expression between normal and tumor tissues (Figure 2A). For further studies, we selected miRNAs that had no less than 2-fold change between the tumor tissues and wildtype tissues (log2 >=1), and had no less than 50 reads per 10 million raw reads in either normal or tumor wing disc samples. Differential expression of the miRNAs is summarized in Figure 2B and displayed in Figure 2C. We found that in tumors 51 mature miRNAs were affected, constituting 10.9% of all mature miRNAs in *D. melanogaster*. Among these affected miRNAs, 28 miRNAs were upregulated and 23 were downregulated in tumor tissues (Figure 2B). Consistent with prior results [12, 13], a known onco-miRNA *bantam* (log2 (*dme-bantam-5p*) ratio =1.9 and log2 (*dme-bantam-3p*) ratio =1.5) and a known tumor suppressor miRNA *let-7* (log2 (*dme-let-7-5p*) ratio =-4.25) were identified as upregulated and downregulated, respectively (Figure 2C). The qRT-PCR analysis of selected miRNAs validated our small RNA-Seq results (Figure 2D and Supplementary Figure 3C). By leveraging the small RNA-seq technique we discovered miRNAs that were upregulated or downregulated in tumors generated by ectopic expression of *lgl-RNAi/Ras*^*V12*^, suggesting their correlation with nTSG defect-induced epithelial tumors.

### Functional analysis reveals tumor-enhancing and tumor-suppressing miRNAs

**Figure 3:**
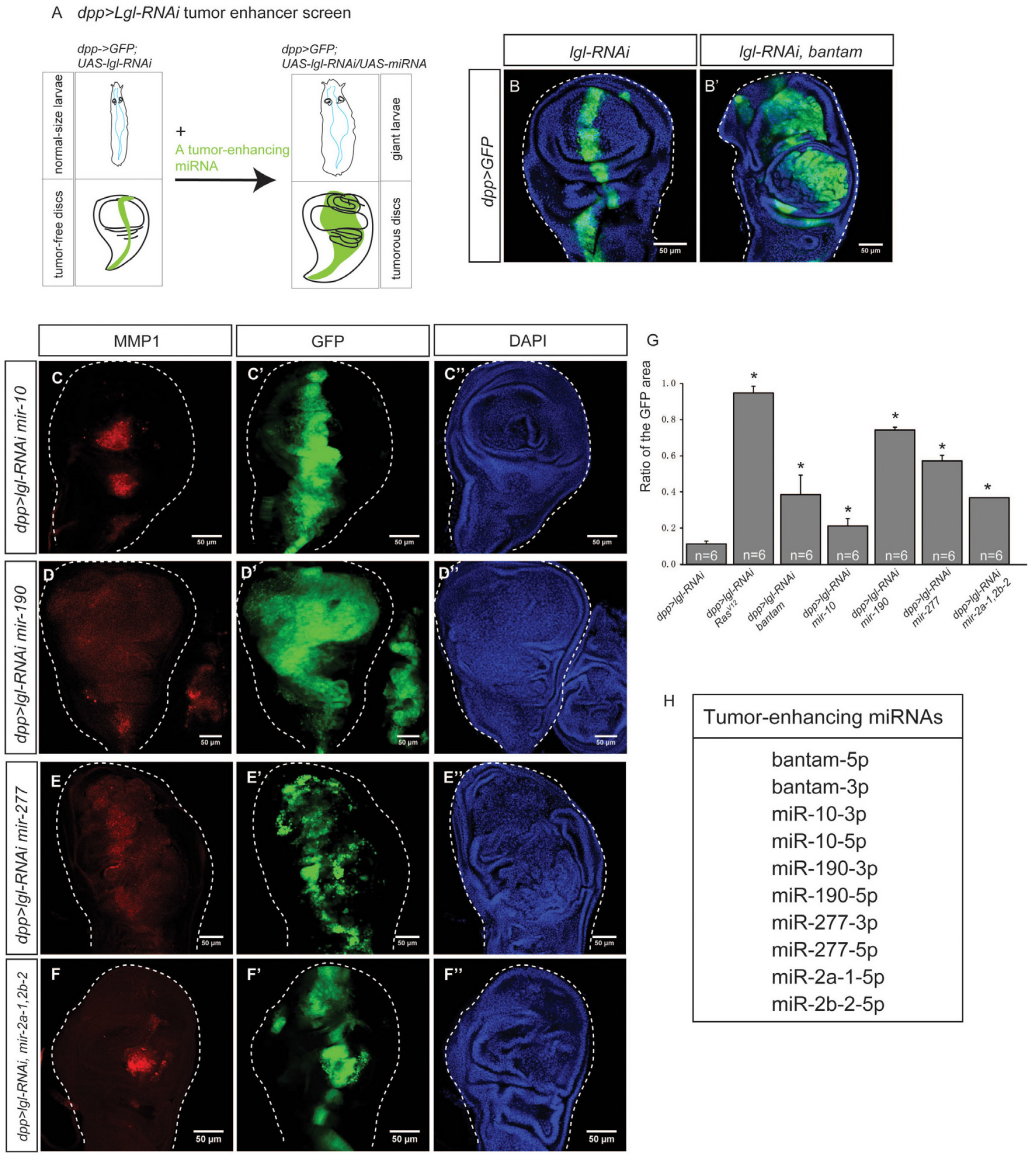
Tumor-enhancing miRNAs collaborate with *dpp>lgl-RNAi* to induce tumorigenesis. **(A)** The *dpp>lgl-RNAi* tumor enhancer screen was used to identify miRNAs that can enhance tumorigenesis. A tumor sensitized fly strain *dpp>lgl-RNAi* was used, in which tumor suppressor lgl was knocked down by *lgl-RNAi*, driven by *dpp-Gal4*. No tumors were observed in the wing imaginal disc and the larva displayed the normal size. A tumor enhancing miRNA can be identified if its overexpression causes the giant larvae phenotype and tumor growth in the wing imaginal disc. **(B-B’)** A known onco-miRNA bantam collaborates with *lgl-RNAi*, driven by *dpp>Gal4* (B), to induce tumors (B’). (C-F’’). Tumor-enhancing miRNAs, mir-10 **(C-C’’)**, mir-190 **(D-D’’)**, mir-277 **(E-E’’)**, and mir-2a-1, 2b-2 **(F-F’’)** were expressed in the *dpp>lgl-RNAi* background individually, and induced tumors. The GFP signal marks gene overexpression and the RFP indicates MMP1, a metastasis marker. Scale=50um. **(G)** Comparison of the width ratio of the GFP area to the wing disc in different genetic combinations. The stars indicate the significance difference exists when *Ras*^*V12*^ or a tumor-enhancing miRNA was expressed. Sample size n=6, p value<0.05. **(H)**. A list of 10 tumor-enhancing miRNAs.

So far, we have identified a list of 28 miRNAs that were upregulated in epithelial tumors. However, overexpression of these miRNAs (*e.g. bantam, miR-190, miR-2a-1, miR-2b-2*) individually does not display tumorigenic phenotypes in the *Drosophila* wing disc (Supplementary Figure 4). To determine whether these miRNAs have a tumor-enhancing role by inducing tumorigenesis synergistically with *lgl* knockdown, we performed a tumor enhancer screen by using a fly strain that expresses *UAS-lgl-RNAi* driven by *dpp-Gal4*. The heterozygous *dpp>lgl-RNAi* flies were viable and had a normal larval size. Their wing imaginal disc maintained normal morphology (Figures 1C–1C’’,3A ), though several signaling pathways was mildly altered (Supplementary Figure 5A–5G’’) and their adult wings had minor morphological defect when raised at 29°C (Supplementary Figure 5H–5H’). The rarely observed homozygous flies, however, showed tumorigenic overgrowth in their wing discs (Supplementary Figure 5G–5G’’) and died at larval stage at 29°C. Moreover, when *bantam* was co-expressed with *dpp>lgl-RNAi*, the larvae had a significant increase in size and the wing discs displayed tumorigenesis and metastasis (Figures 3A, and 3B–3B’). Therefore, this tumor-sensitized fly strain is suitable for a tumor enhancer screen for identifying genes that can induce tumorigenesis.

**Figure 4:**
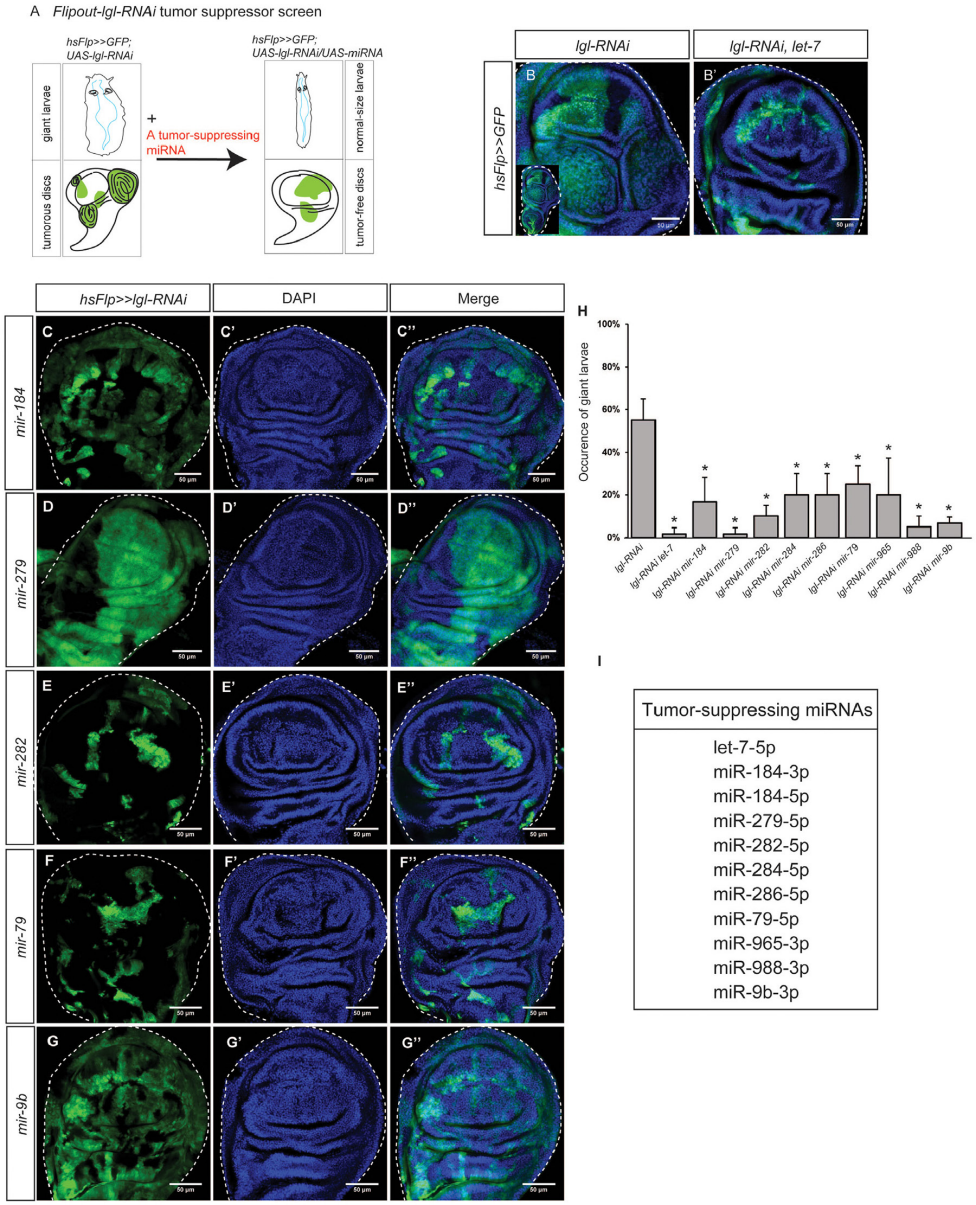
Tumor-suppressing miRNAs inhibit *lgl-RNAi* induced epithelial tumors. **(A)** The *Flipout-lgl-RNAi* tumor suppressor screen was used to identify tumor suppressing miRNAs. Tumor suppressor lgl was knocked down by *lgl-RNAi* driven by flipout actin-Gal4. A tumor-suppressing miRNA can be identified if its overexpression represses tumorous phenotypes and restores tissue organization. **(B-B’)** Tumors induced by *lgl-RNAi*, driven by flipout actin-Gal4 were observed in the wing discs (B). The enlarged whole disc is shown in the inset. A known tumor suppressor miRNA *let-7* inhibits *lgl-RNAi*-induced tumorigenesis (B’). **(C-G’’)** Tumor-suppressing miRNAs, including mir-184 (C-C’’), mir-279 (D-D’’), mir-282 (E-E’’), mir-79 (F-F’’), and mir-9b (G-G’’), were expressed in the *hsFlp>>lgl-RNAi* background individually. The GFP signal shows gene overexpression. Scale=50um. **(H)** Comparison of the giant larva occurrence in different genetic combinations. The stars indicate the significance difference exists when a tumor-suppressing miRNA was expressed. Sample size n=3, p-value<0.05. **(I)** A list of 11 tumor-suppressing miRNAs.

**Figure 5:**
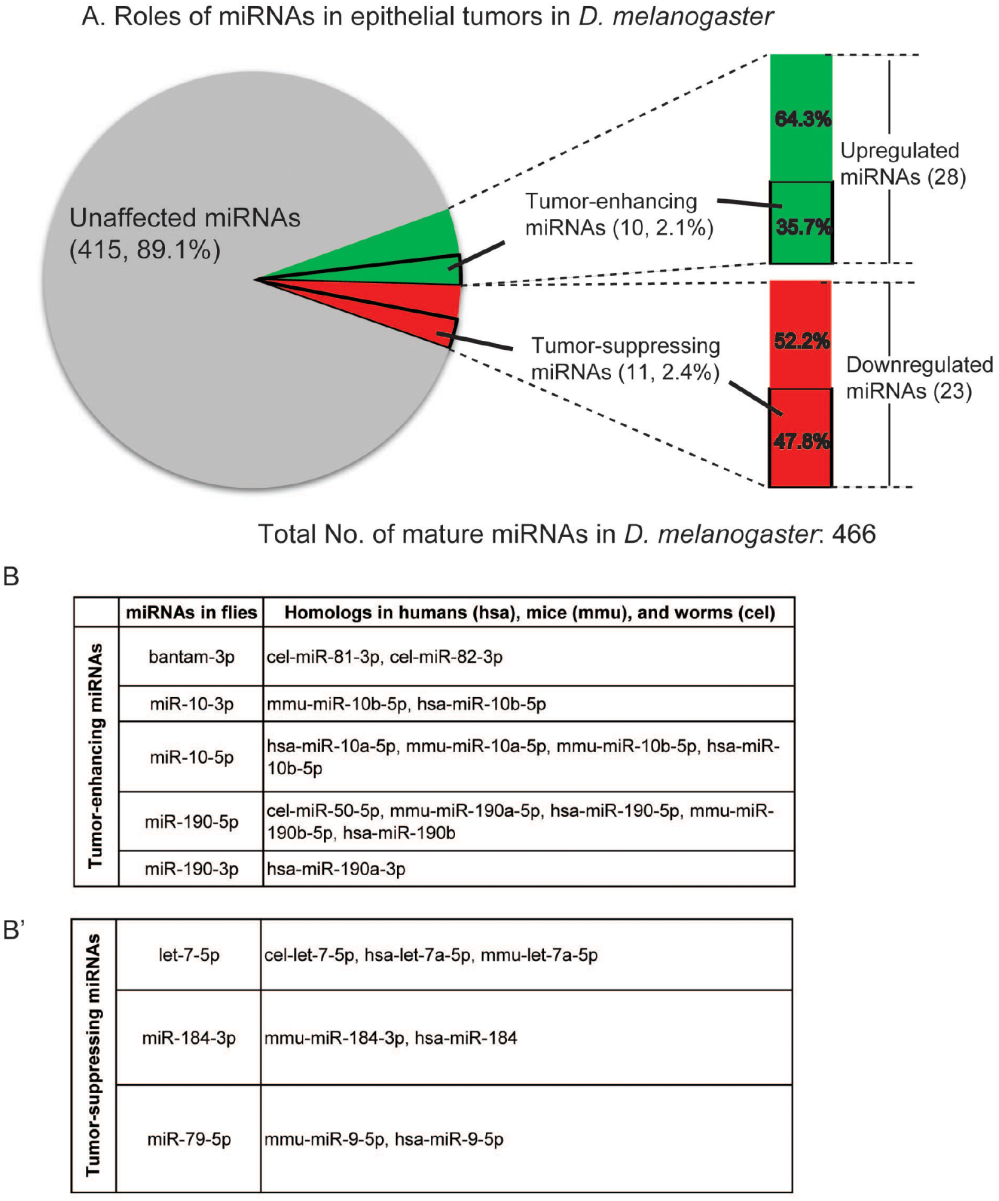
Conservation of the tumor-implicated miRNAs in humans. **(A)** Pie chart showing the roles of miRNAs in epithelial tumors: 28 miRNAs were upregulated in nTSG defect-induced tumors, 11 of which were identified as tumor-enhancing miRNAs; 23 miRNAs were downregulated in these tumors, and 11 of them were identified as tumor-suppressing miRNAs. **(B-B’)** Tumor-enhancing and tumor-suppressing miRNAs are conserved in humans, mice, and worms. Four tumor-enhancing (B) and three tumor-suppressing (B’) miRNAs have homologs in humans.

**Figure 6:**
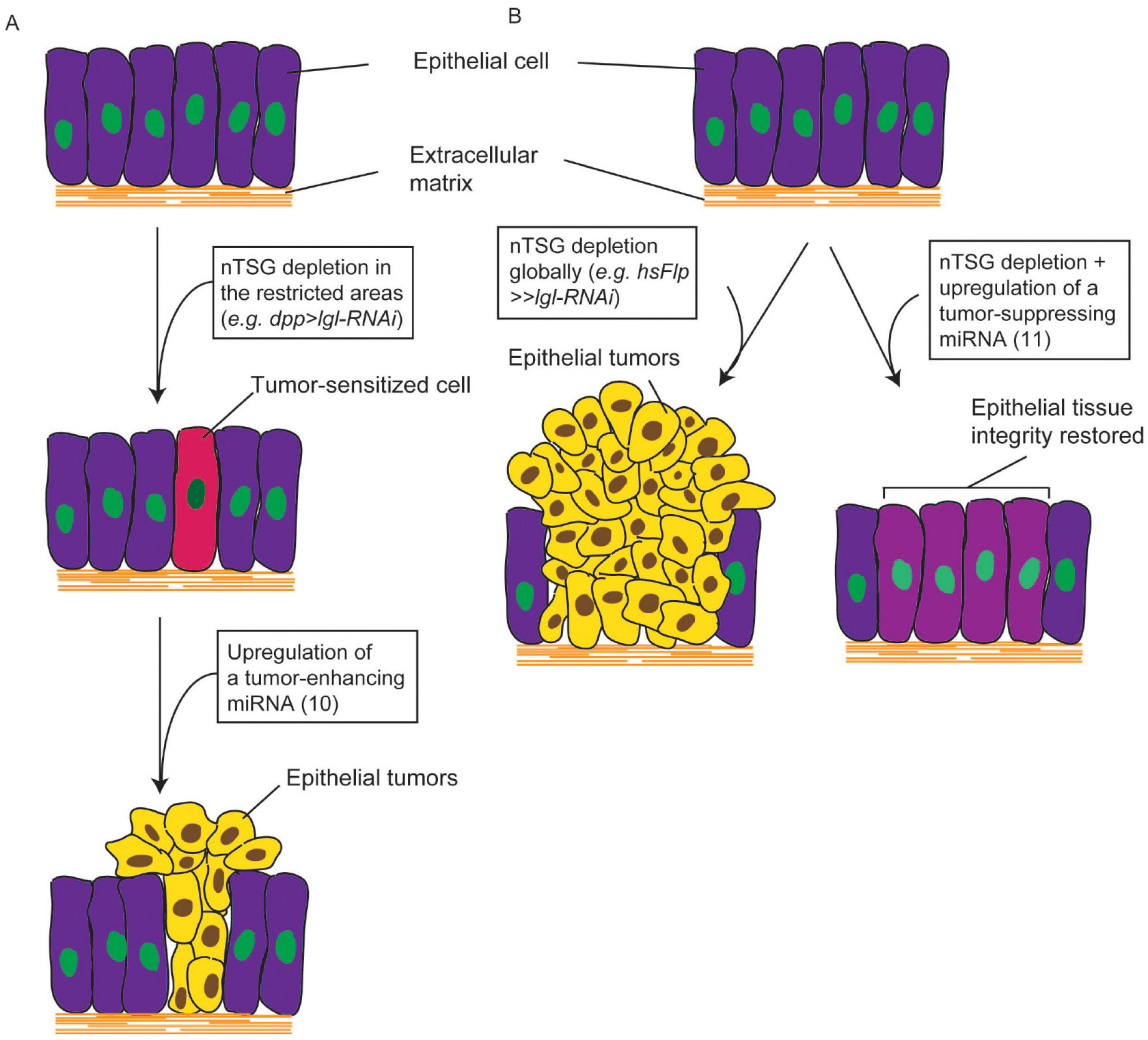
miRNAs are involved in Drosophila nTSG defect-induced epithelial tumors. **(A)** Depletion of an nTSG in the restricted areas in epithelia (*e.g. dpp>lgl-RNAi*) does not induce tumorigenesis but sensitizes the cells. Overexpression of a tumor-enhancing miRNA promotes cell proliferation and tissue growth, and transforms these cells to form epithelial tumors. **(B)** Epithelial tumors form when an nTSG is depleted globally (*e.g. hsFlp>>lgl-RNAi*). Activation of a tumor-suppressing miRNA inhibits tumor growth and restores epithelial tissue integrity.

Using the *dpp>lgl-RNAi* tumor enhancer screen, we tested 28 (available fly stocks, Supplementary Table 2) miRNAs upregulated in the *lgl-RNAi*-induced tumors, and found that 10 of them induced tumorigenic overgrowth and upregulation of MMP1, indicating a metastatic potential for the tumors (Figure 3C–3F’’). The results suggest miRNAs’ ability to enhance tumorigenesis through collaborating with nTSG mutations. As such, we named them “tumor-enhancing (TE) miRNAs.” To quantify the effects of overexpression of TE miRNAs on tumor growth, we calculated the width ratio of the GFP area to the entire wing disc. When *Ras*^*V12*^ was overexpressed with *lgl-RNAi*, the width ratio significantly increased (Figure 3G). Similarly, overexpression of a TE miRNA with *lgl-RNAi* significantly increased the width ratio (Figure 3G, n-6, p-value < 0.05), suggesting the TE miRNAs can collaborate with *lgl-RNAi* to significantly promote cell proliferation and tissue growth. Through this screen, we also identified 18 miRNAs that were upregulated in epithelial tumors but did not induce tumors in the tumor-sensitized background individually, suggesting that their upregulation was dispensable to tumorigenesis and might be the result of epithelial tumors. In summary, we list 10 TE miRNAs in Figure 3H. Using microRNA.org, we identified miRNAs that target *lgl* mRNA, and found none of the 16 miRNAs are TE miRNAs (Supplementary Table 3). This rules out the possibility that these TE miRNAs directly suppress *lgl* expression.

To further test the roles of TE miRNAs in tumorigenesis, we suppressed their expression in epithelial tumors by expressing their sponge forms, which bind with miRNAs to attenuate their effects [27]. We found that expression of the sponge lines inhibits tumors (Supplementary Figure 6), suggesting that these TE miRNAs are required for primary epithelial tumor formation. In addition, we did not observe tumors when these TE miRNAs were coexpressed with Ras^V12^ (Supplementary Figure 7), ruling out the possibility that TE miRNAs may synergize with Ras^V12^ to induce tumors.

From the RNA-Seq analysis, we also identified a list of 23 downregulated miRNAs (Figure 2B and 2C). To determine whether these downregulated miRNAs are actively involved in tumorigenesis, we overexpressed them in nTSG defect-induced tumors. Five days after *lgl-RNAi* induction by the Flip-out UAS/Gal4 technique, the larvae showed the giant larva phenotype and wing discs displayed overgrowth and epithelial tumors (Figure 4A). When we expressed a known tumor suppressor *let-7* in *lgl-RNAi*-induced tumors, the normal disc morphology was restored and tumorigenesis was suppressed (Figure 4B–4B’), consistent with the tumor suppressor role of *let-7*. The results therefore indicate that conducting a *Flipout-lgl-RNAi* tumor suppressor screen of genes of interest allows us to identify the ones that are potent to suppress nTSG defect-induced tumorigenesis.

Using this method, we tested 22 downregulated miRNAs (available fly stocks in Supplementary Table 2), and found that 11 of them could suppress *lgl-RNAi*-induced tumorigenesis, restoring the normal tissue organization (Figure 4C–4G’’ and Supplementary Figure 8). Because their addition inhibited nTSG defect-induced tumorigenesis, we named these miRNAs “tumor-suppressing (TS) miRNAs.” To quantify the tumor suppression effects of miRNA overexpression, we measured the occurrence of the giant larva phenotype as an indicator of tumor suppression effectiveness. We confirmed that co-expression of *let-7* in the *lgl-RNAi* expressing clones strongly suppressed the giant larvae phenotype (Figure 4H). We found that, similarly, the TS miRNAs significantly decreased the giant larva occurrence in the *hsFlp>>lgl-RNAi* background (Figure 4H, n=10, p-value < 0.05). In contrast, overexpression of other 12 downregulated miRNAs failed to inhibit tumorigenesis individually, suggesting that their low expression levels were less critical to epithelial tumors. In addition, none of the TS miRNAs target the oncogene *Ras* (Supplementary Table 3), suggesting these TS miRNAs do not directly inhibit Ras overexpression. To summarize, we list 11 TS miRNAs in Figure 4I.

A global decrease in miRNA levels is often observed in human cancers [28]. Indeed, total reads of miRNAs were significantly fewer in the tumorous wing disc (averagely 6,053,895 reads per 10 million raw reads) than in the wildtype wing disc (averagely 8,994,225 reads per 10 million raw reads, three samples, p-value <0.05). To rule out the possibility that miRNAs may generally interfere with tumorigenesis, we disrupted miRNA biogenesis by knocking down Dicer-1 or pasha [4, 6]. Reducing miRNA levels globally did not induce tumors in *dpp>lgl-RNAi* (Supplementary Figure 9), suggesting the roles of miRNAs in tumors are miRNA specific. Taken together, our results show that TE miRNAs are upregulated in nTSG defect-induced epithelial tumors and their overexpression further promotes tumorigenesis, whereas TS miRNAs are downregulated in nTSG defect-induced epithelial tumors and their overexpression inhibits tumorigenesis.

In addition, we identified target genes of the TE and TS miRNAs (Supplementary Table 3), and found many target genes that are related to critical signaling pathways. For example, TE miRNA miR-190 can target Socs36E, a negative regulator of JAK/STAT signaling [29], suggesting miR-190 may enhance tumorigenesis through activating JAK/STAT signaling. In another example, several TS miRNAs target genes involved in cytoskeletal motility and cell migration, such as RhoGAP68F and RhoGAP100F [30], suggesting they may inhibit tumor invasion.

### Tumor-implicated miRNAs are conserved in humans

Therefore, we summarize our findings in Figure 5A. Combining RNA-Seq and functional genetics, we found 28 upregulated miRNAs and identified 10 TE miRNAs; we also found 23 downregulated miRNAs and identified 11 TS miRNAs. Because all mammalian miRNA families are represented in *Drosophila* [3], it is very likely that tumor-implicated miRNAs we identified in flies have similar functions in humans. Using the miRNA gene family search function in the miRbase database, we found that 26 *Drosophila* precursor miRNAs have human homologs (Supplementary Table 4). To investigate whether these tumor-implicated *Drosophila* miRNAs may be conserved in different organisms, we conducted the miRNA sequence and identified five TE miRNAs and three TS miRNAs conserved in other organisms (Figure 5B–5B’). More interestingly, all the human homologs of these miRNAs have been reported to be involved in various human cancers (Figure 5B–5B’).

For example, *miR-10*, a TE miRNA identified in this study, is highly conserved in metazoans and regulates the Hox gene, indicating its critical role during development [31]. In humans, *mir-10* is located in a region amplified in melanoma and breast cancer, and is highly upregulated in a broad range of human cancers, such as glioblastoma, pancreatic cancer, colon cancer, and breast cancer [32, 33]. On the TS miRNA side, downregulation of *miR-184* has been found to promote human malignant glioma by regulating its target *SND1*, a multifunctional nuclease that is overexpressed in multiple cancers [34]. The involvement of human homologs of TE and TS miRNAs in cancers is shown in Figure 5B and 5B’.

## DISCUSSION

The data presented here reveal an important classification of *Drosophila* miRNAs based on their relationship with epithelial tumors (Figure 5A). Through an RNA-Seq analysis, we identified 51 tumor-implicated miRNAs, whose expression levels were significantly altered in nTSG defect-induced epithelial tumorigenesis. These miRNAs were functionally examined for their causal relationship with *Drosophila* tumors using sensitized genetic backgrounds: *dpp>lgl-RNAi* for a tumor enhancer screen (Figure 3A) and *Flipout>>lgl-RNAi* for a tumor suppressor screen (Figure 4A). Of note, while tumors could form at 25°C, all crosses were performed at 29°C to enhance tumor growth and increase tissue’s sensitivity to tumorigenic perturbation. Among them are 10 TE miRNAs, in which four are conserved in humans, and 11 TS miRNAs, in which three are conserved in humans. In these epithelial tumors, TE miRNAs transform tumor-sensitized cells and promote tumor growth (Figure 6A), whereas TS miRNAs may inhibit nTSG defect-induced tumors and restore epithelial tissue integrity (Figure 6B). These two platforms provide convenient tools to uncover small regulatory RNAs that have mild tumor-enhancing or tumor-suppressing roles in epithelial tumors. It is also interesting to confirm that overexpression of TE miRNAs by the tumor enhancer screen matches their elevated expression levels in *lgl* defect-induced tumors, and overexpression of TS miRNAs by the tumor suppressor screen restores their expression levels in the wildtype to inhibit tumorigenesis.

We show that four TE miRNAs and three TS miRNAs have human homologs (Figure 5B–5B’). Based on the gene family analysis, we found 52 conserved *Drosophila* precursor miRNAs (Supplementary Table 4), seven of which are involved in epithelial tumorigenesis. Their critical roles in human cancers have been reported by prior research. In addition to *miR-10* discussed above, *miR-190* has also been mechanistically involved in various human cancers. Overexpression of *miR-190* enhances proliferation and malignant transformation through activation of Akt signaling [35]. On the TS miRNA side, *let-7* has been found as a tumor suppressor in human lung cancer [13, 36] and its target genes including oncogenes *RAS* and *HMGA2* [37, 38]. Interestingly, *miR-79*, a TS miRNA, is a *miR-9* gene family member, which is upregulated in breast cancer [39]. It will be interesting to determine whether *miR-79* can also act as a tumor suppressor in other types of human cancer.

The combined RNA-Seq and functional screens have missed some conserved miRNAs that show a role in either tumor promotion or suppression in human cancers. For example, *miR-34*, which is known for its tumor suppressor role in cancers [40], did not show a high enough change to meet the two-fold cutoff between the tumor and normal discs in the RNA-Seq assay. The change levels (log2) of *miR-34-3p* and *miR-34-5p* were 0.32 and −0.12, neither of which substantiates functional genetic studies. Since we only analyzed wing imaginal disc derived tumors, we cannot rule out the possibility that *miR-34* acts as a tumor suppressor in a different tissue.

Another example is *miR-1*, which acts as a tumor suppressor in various human cancers, including head and neck squamous cell carcinoma, lung cancer, and glioblastoma [41]. It was not identified as either TE or TS miRNA in our study, because log2 (*miR-1-3p*) = −0.76, whereas *miR-1-5p* only had two reads and 0.33 read on average in the wildtype and epithelial tumors. Although the expression of *miR-1* is low in the control and tumor discs, it could enhance nTSG defect-induced tumorigenesis when overexpressed (Supplementary Figure 10A–10A’’).

In addition to *miR-1*, several other miRNAs that had very few reads (fewer than 50 reads per 10 million raw reads) in both the tumor and wildtype tissue, could enhance or suppress tumorigenesis when overexpressed. For example, *miR-315-5p* enhanced tumors in *dpp>lgl-RNAi*, but had 3.0 reads and 11.7 reads on average in normal and tumor tissues, respectively (Supplementary Figure 10B–10B’’ and Supplementary Table 1); *miR-285-3p* suppressed *hsFlp>>lgl-RNAi* tumors, but had 3.7 reads and 0.67 reads on average in normal and tumor tissues, respectively (Supplementary Figure 10E–10E’’ and Supplementary Table 1). Similarly, *miR-315, miR-6-1, 6-2, 6-3*, and *miR-981* could individually enhance epithelial tumors in the *dpp>lgl-RNAi* background (Supplementary Figure 10B–10D’’), whereas *miR-954* and *miR-955* suppress *hsFlp>>lgl-RNAi*-induced tumorigenesis (Supplementary Figure 10E–10G’’). The results suggest that though these miRNAs can affect tumorigenesis when overexpressed, they had extremely low expression levels in the wing imaginal disc epithelial cells and are unlikely contributors to *lgl* defect-induced epithelial tumors. Since miRNAs show dynamic spatial and temporal patterns during organ development [42], the relationship between miRNAs and tumorigenesis can be tissue specific and context dependent. These miRNAs may have higher levels of expression in other tissues as well as tumors derived from those tissues, and thus have a more relevant role in related tumorigenesis.

Another caveat in our analysis is that we expressed miRNAs one at a time to test their ability to enhance or suppress tumorigenesis; however, we did not rule out the possibility that miRNAs may cooperate to actively contribute to tumorigenesis. In fact, complex diseases are often affected by several miRNAs rather than a single miRNA. For example, *miR-125a, miR-125b*, and *miR-205* have been reported to functionally cooperate to downregulate the erbB receptor tyrosine kinase family components erbB2/erbB3 in breast cancer cells [43]. More work needs to be done to delineate miRNA synergism, specifically to understand whether multiple miRNAs collectively can enhance or suppress tumorigenesis.

How tumorigenesis affects miRNA expression is a long-standing question. Several mechanisms have been proposed to address this question. First, genetic abnormalities, including chromosomal rearrangements, deletions and mutations can alter miRNAs. In humans, more than half of miRNAs are frequently altered in cancer, of which 65 miRNAs are in the loss-of-heterozygosity regions where the majority of tumor-suppressor genes are located [11]. Second, miRNAs in tumors maybe altered by epigenetic aberrations, such as DNA hypermethylation of tumor-suppressor genes. An increase in the methylation of many tumor-suppressor miRNAs further allows overexpression of the oncogenic targets [44]. The third mechanism is transcriptional control. For example, the *myc* oncogene can transcriptionally activate the *mir-17/92* cluster [45]. Lastly, posttranscriptional control of miRNA biogenesis may also have an impact on mature miRNA levels. Tumorigenesis may affect many components in the miRNA biogenesis pathway, leading to dysregulation of miRNA expression [46]. The tumor-implicated miRNAs identified in our study may be differentially regulated through one or more of these transcriptional or post-transcriptional mechanisms during tumorigenesis, and they further lead to functionally relevant downstream consequences. Reversion of miRNA expression to normal levels shows practically favorable outcomes [47]. For example, a utility patent was granted on using *miR-10* and its relevant targets in assessing and treating the condition of a patient [48]. On the other hand, TS miRNAs identified through our study may be supplied to tumors to restore their normal cellular levels, as they are usually underexpressed in cancer. In this case, miRNA replacement strategies have been developed [49]. To effectively administer miRNA reversion or miRNA replacement therapies, a critical step is to identify miRNAs that actively contribute to the tumor, which are TE and TS miRNAs. In this sense, the identification of evolutionarily conserved TE and TS miRNAs in this study helps to provide potentially important therapeutic targets for cancer treatment.

## MATERIALS AND METHODS

### Fly stocks and genetics

All flies were maintained at 25°C. For the *dpp>lgl-RNAi* tumor enhancer screen, *dpp>lgl-RNAi* was crossed to flies that carry an RNAi or an overexpression construct under the UAS promoter. Their progeny flies were cultured in 29°C for five to seven days before dissection. For the *Flipout>>lgl-RNAi* tumor suppressor screen, *hsFLP; actin>y>Gal4* was crossed to flies that carry an RNAi or an overexpression construct under the UAS promoter. Two days after egg deposition (AED), larval progeny were heat shocked at 37°C for 30 minutes. The progeny flies were kept at 25 °C for at least five days before dissection. A construct of UAS-GFP (or RFP), which expresses fluorescent green (or red) protein, was always included in the cross.

The following fly stocks were used in this study:

*dpp-Gal4* (BL7007)

dpp-Gal4, UAS-GFP; UAS-lgl-RNAi, UAS-Dicer 2/T(2:3)

hsFlp; actin>y>Gal4, UAS-GFP; UAS-lgl-RNAi, UAS-Dicer-2

All miRNA fly stocks are listed in Supplementary Table 2.

### Immunocytochemistry

Immunocytochemistry was carried out as described previously [50]. The following antibodies were used: mouse anti-MMP1 (1:1:1 mixture of 3B8, 3A6 and 5H7 were diluted 1:40, DSHB), and secondary antibodies Alexa 488, 546, or 633 (1:500) (Invitrogen). Images were captured on a Zeiss LSM-800 confocal microscope. Images were processed and arranged in Image J and Adobe Illustrator.

### Small RNA library preparation and analysis

Approximately 200 wing imaginal discs from wild-type (*w1118*) and tumorous environments were dissected. Small RNA libraries were prepared per instructions for Illumina TrueSeq Small RNA sample prep kit and some modifications, as describe in [51]. For sequencing, Illumina HiSeq 2500 system was used, and samples were under single end, 50-base pair conditions. Reads were demultiplexed and indexes removed with CASAVA v1.8.2 (Illumina). The 3’ adapter sequences were trimmed and reads with more than 10% having a Sanger quality score of less than 25 were discarded with the FastX-toolkit (http://hannonlab.cshl.edu/fastx_toolkit/). Briefly, after clipping the Illumina 3′-adapter sequence (TGGAATTCTCGGGTGCCAAGGAA CTCCAGTCAC) using cutadapt (cutadapt.readthedocs.org/en/latest), the small RNA reads that passed quality control through removal of low-complexity or low-quality sequenced reads using sickle (github.com/najoshi/sickle), and the length filter (18∼35-nt) were mapped to the miRNA sequences in *Drosophila melanogaster* release 21 assembly [26] with Bowtie2 [52], allowing two mismatches. The per-base coverage was calculated per 10 million raw reads with BEDTools [53].

Average expression levels of miRNAs in tumorous and wildtype environments from three biological samples were compared. We only selected miRNAs that had at least 50 average reads in either wildtype wing disc samples or tumorous samples for analysis. Among them, the miRNAs with no less than two-fold changes were defined as significantly differentially expressed in two environments (An absolute value of log2 ratio>1 was used as the threshold to determine the significance of expression difference.

### Length profile and relative abundance of miRNAs

FASTX (http://hannonlab.cshl.edu/fastx_toolkit/) was used to remove adapter sequence from raw reads. Reads without adapter, shorter than 18 nt or mapped to rRNAs were filtered out. The miRNAs were identified by miRDeep2 [54]. miRNA length statistics were carried out as previously described [55]. The miRNA read abundance were calculated by miRDeep2 and normalized with DESeq [56].

### Quantitative real-time PCR

Total RNA from the Drosophila wing imaginal disc was isolated using Trizol Reagent and miScript (Qiagen) per the manufacturer’s instructions. One microgram of total RNA was reverse-transcribed in 20 μl of reaction mixture containing Superscript II reverse transcriptase (Invitrogen) and oligo (dT) 12–18 primer per the protocol for the Superscript II first-strand cDNA synthesis system. One microliter cDNA (reverse transcribed from 50 ng of RNA) was subjected to quantitative real-time PCR (in 25 μl reaction volume) by using primers specific to each miRNA (primer sequences are listed in Supplementary Table 5) and cDNA templates were amplified using the Platinum SYBR Green qPCR SuperMix UDG kit, per the manufacturer’s instructions (Invitrogen). PCR conditions were: 95 C for 10 min; 40 cycles of 95 C for 30s, 58 C for 15s, and 68 C for 45s. Real-time PCR was performed using the ABI 7500 Thermocycler (Applied Biosystems), and results were analyzed using SDS version 2.1 software (Austin Biodiversity Web site gallery). Data analysis was done using the 2^−ΔΔCT^ method for relative quantification. Calculated expression values of cDNA samples were normalized to 5S rRNA.miRNA specific primers are listed in Supplementary Table 5.

### Identifying relevance of the mRNA and miRNA

To find miRNAs that can target *lgl* or *Ras,* *microRNA.org* [57, 58] was used. Display options were set to “view target sites of conserved miRNAs with good mirSVR scores.” To find target genes of TE and TS miRNAs, www.targetscan.org/fly_12/ [59, 60] was used.

### Identifying homologs of miRNAs in other species

The sequence of tumor-enhancing (TE) and tumor-suppressing (TS) miRNAs was found in miRBase.org [26], and was entered to search homologs in miRbase by sequence. The parameters are as follows: Search sequences: Mature miRNAs, Search method: BLASTN, E-value cutoff: 1, Maximum no. of hits: 100, and Show results only from specific organisms: human, mouse, worm, and fly.

## SUPPLEMENTARY MATERIALS FIGURES AND TABLES












