# Supplementary material for: Systematic analysis reveals tumor-enhancing and -suppressing microRNAs in Drosophila epithelial tumors
Source: Oncotarget. 2017 Nov 1;8(65):108825–39. doi: 10.18632/oncotarget.22226 (PMC5752484; doi:10.18632/oncotarget.22226)
Supplement: Supplementary file 1 [file oncotarget-08-108825-s001.pdf]

Systematic analysis reveals tumor-enhancing and -suppressing microRNAs in *Drosophila* epithelial tumors

SUPPLEMENTARY MATERIALS

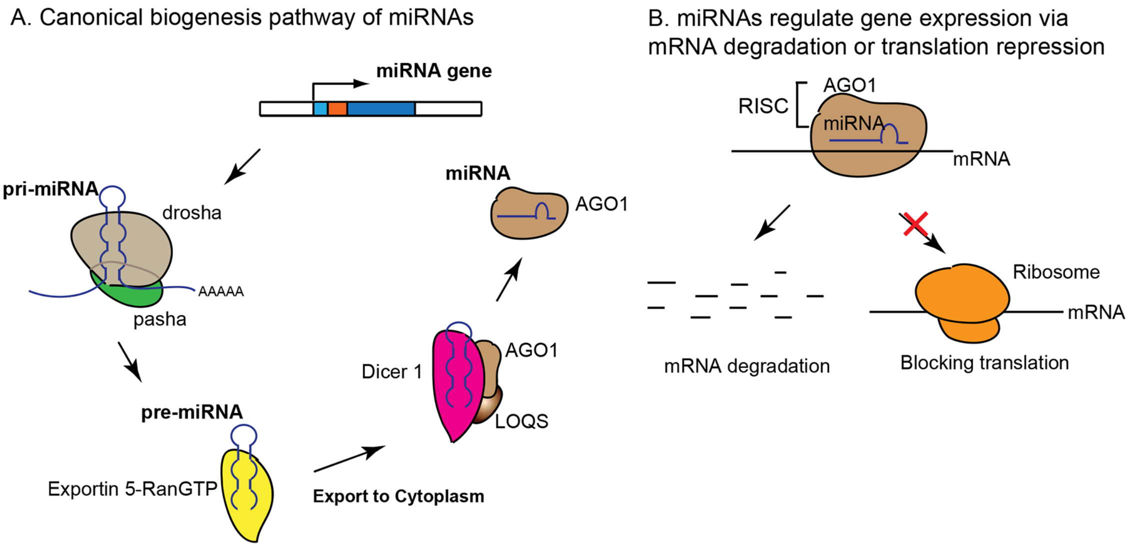

Supplementary Figure 1: Biogenesis and functions of miRNAs in *Drosophila* **(A)** The canonical miRNA biogenesis pathway starts from miRNA genes in the genome, which are transcribed to primary miRNAs (pri-miRNAs). Pri-miRNAs are processed by Drosha and Pasha to form precursor miRNAs (pre-miRNAs) with 70-nt in length. Pre-miRNAs are exported to cytoplasm and further processed by Dicer1 to form mature miRNAs, with approximately 22-nt in length. **(B)** After being loaded onto AGO1 to form RISC, miRNAs affect target gene expression via mRNA degradation or translation repression.

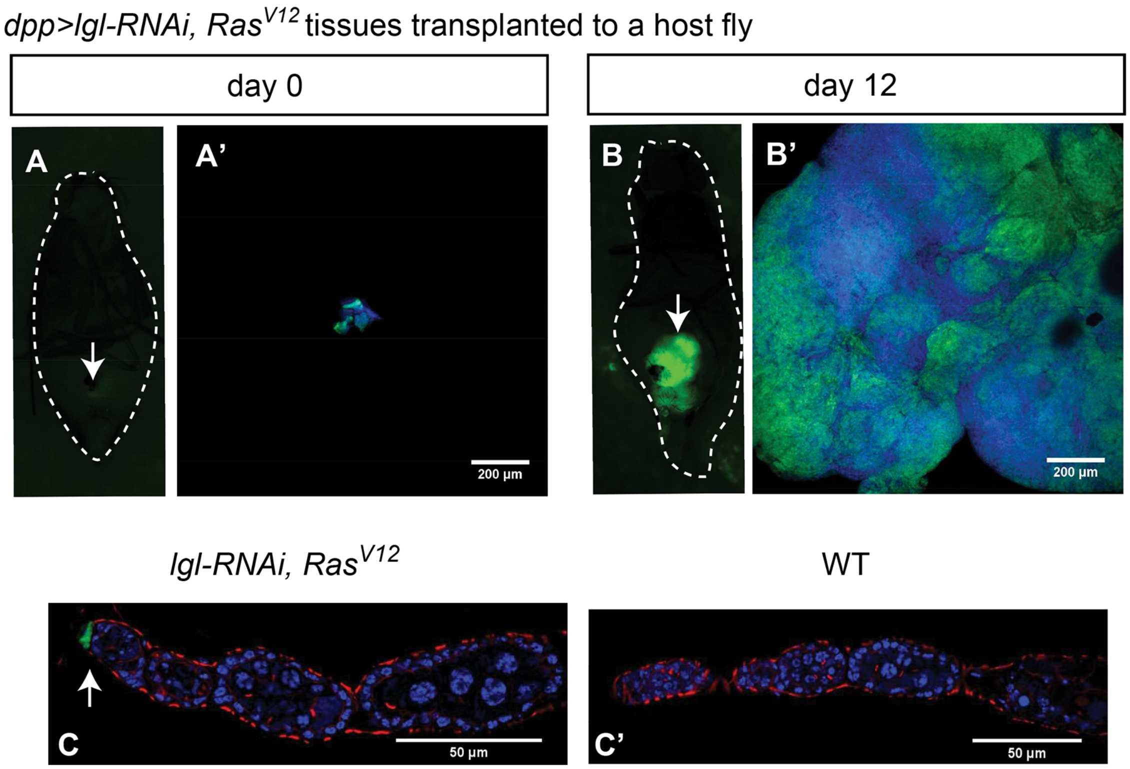

Supplementary Figure 2: *lgl-RNAi, Ras^{V12}* tumorous tissues overgrow and metastasize in host flies **(A-B').** The abdomen section of a female host fly with injected tumorous tissues at day zero (A) and day 12 (B) after transplantation. The tumorous tissues were induced by *dpp>lgl-RNAi, Ras^{V12}*, marked by GFP. The tissues were dissected and shown in A' and B'. White dotted lines indicate entire adult flies. Arrows show the transplanted tissues. **(C-C')** Ovaries of flies hosting *lgl-RNAi, Ras^{V12}* tumorous tissues (C) and the wildtype tissues (C') were dissected. The arrow indicates metastasis by tumorous tissue, marked by GFP. Scale = 50um.

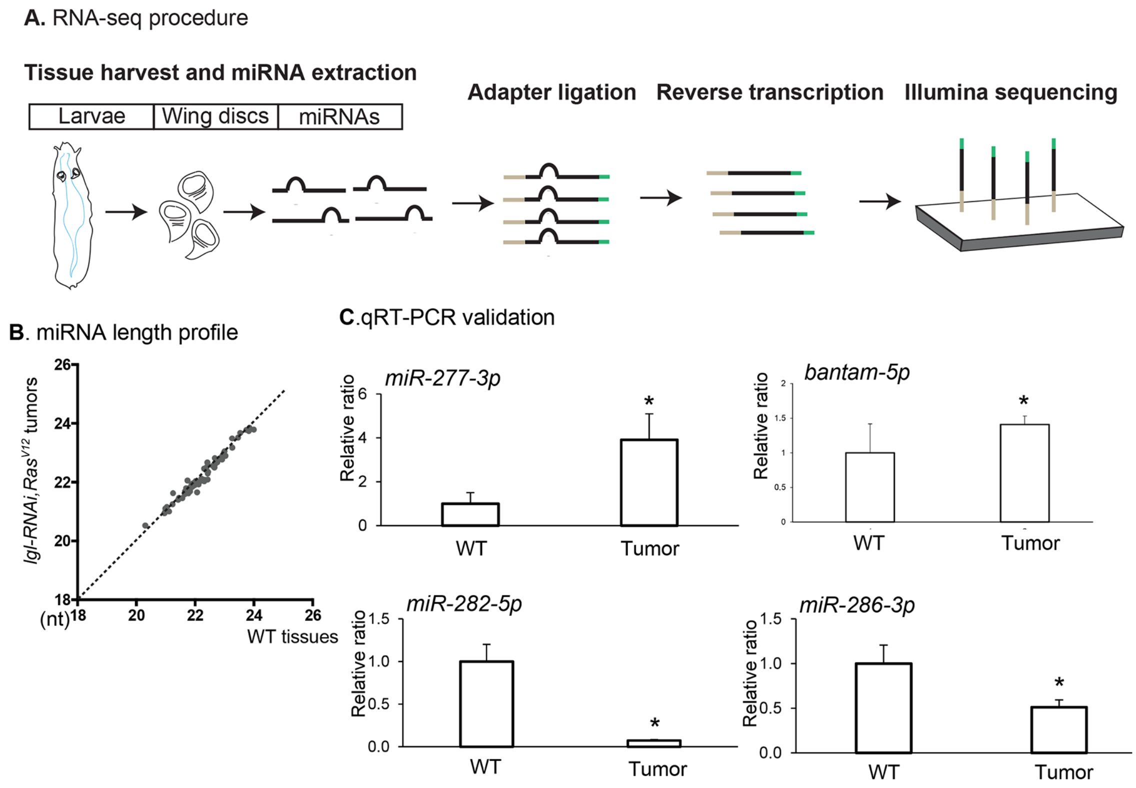

Supplementary Figure 3: The RNA-seq procedure, miRNA length profile, and additional qRT-PCR validation **(A)** The RNA-seq procedure is illustrated. Detailed description in the Materials and Methods section. **(B)** Scatter plot for miRNA length profile according to miRDeep1 output, showing a comparison of the length (nt) of the 466 *D. melanogaster* miRNAs in *lgl-RNAi, Ras*^{V12} tumor tissues and wildtype (WT) tissues. **(C)** Quantitative RT-PCR was conducted on miR-277, bantam, miR-282, and miR-286 to confirm their miRNA level changes. Stars indicate the significant differences. Sample size n=3, p-value < 0.05.

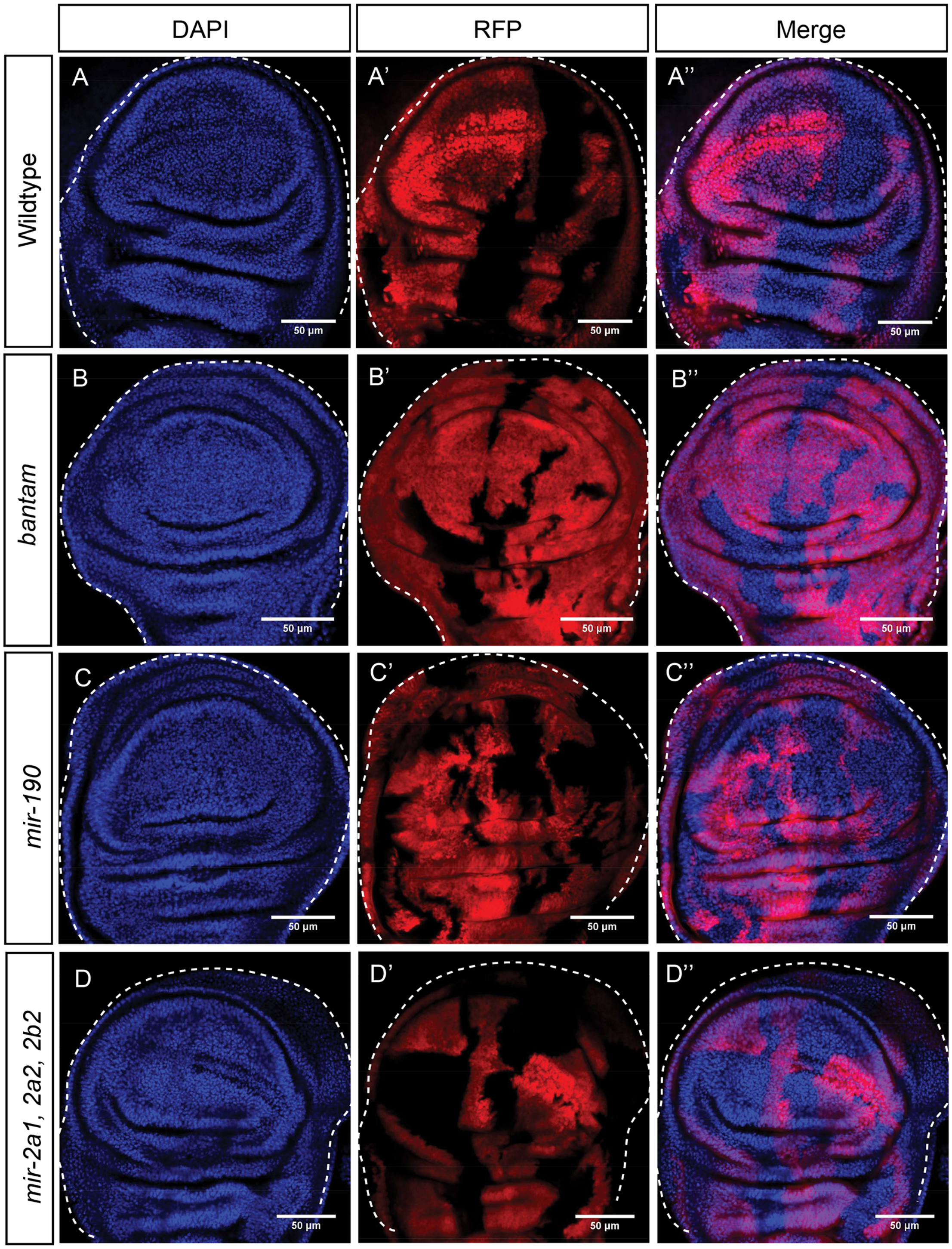

Supplementary Figure 4: Overexpression of tumor-implicated miRNAs does not induce tumors **(A-D")** Overexpression of wildtype (A-A"), bantam (B-B"), mir-190 (C-C"), and mir-2a1,2a2,2b2 (D-D"), driven by flipout-actin-Gal4 in the wing disc. Ectopic gene expressing cells are marked by RFP. Scale=50um.

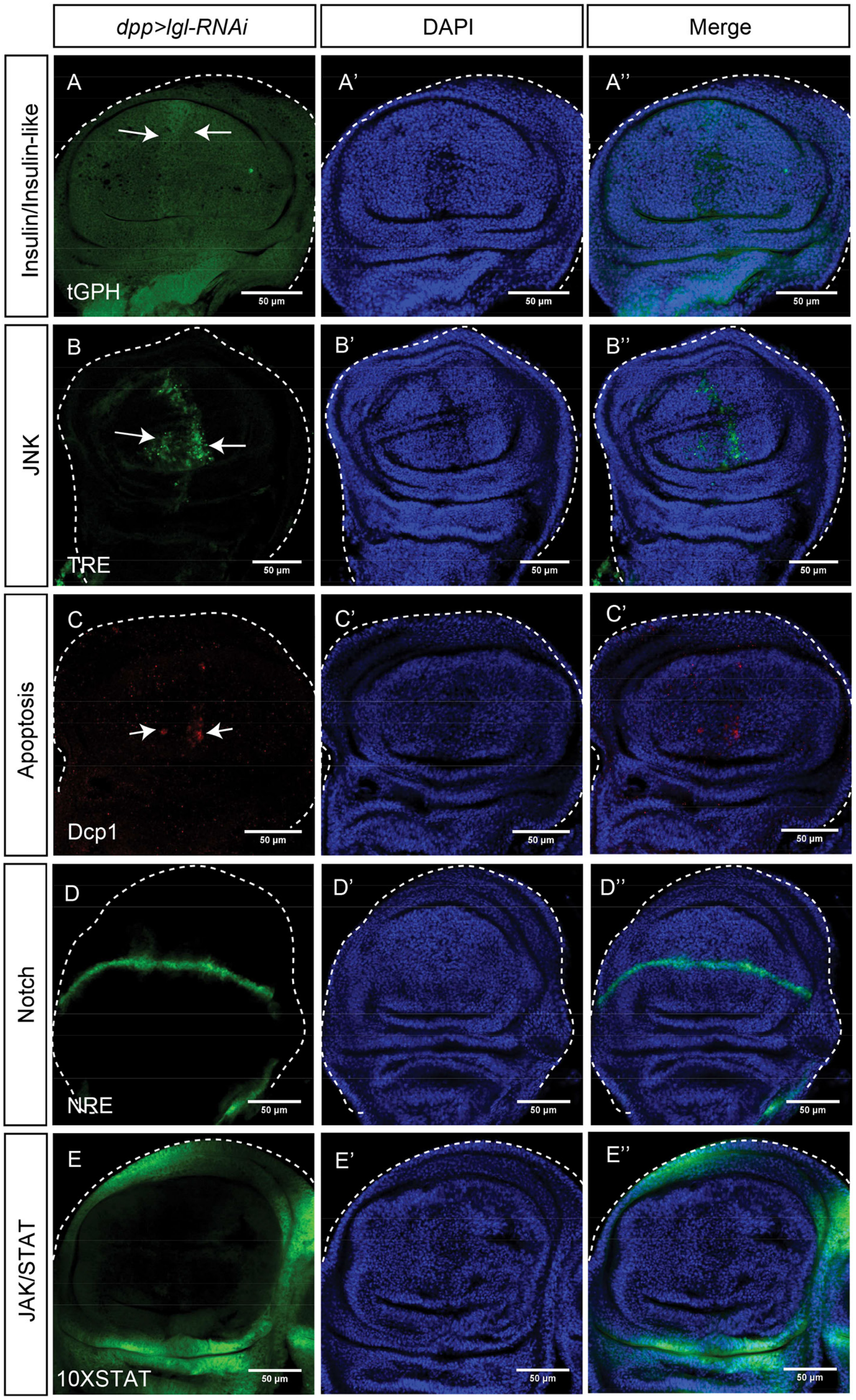
dpp>lgl-RNAi
DAPI
Merge
Insulin/Insulin-like
JNK
Apoptosis
Notch
JAK/STAT
A
A'
A''
tGPH
B
B'
B''
TRE
C
C'
C'
Dcp1
D
D'
D''
NRE
E
E'
E''
10XSTAT
50 μm

(*Contiinued*)

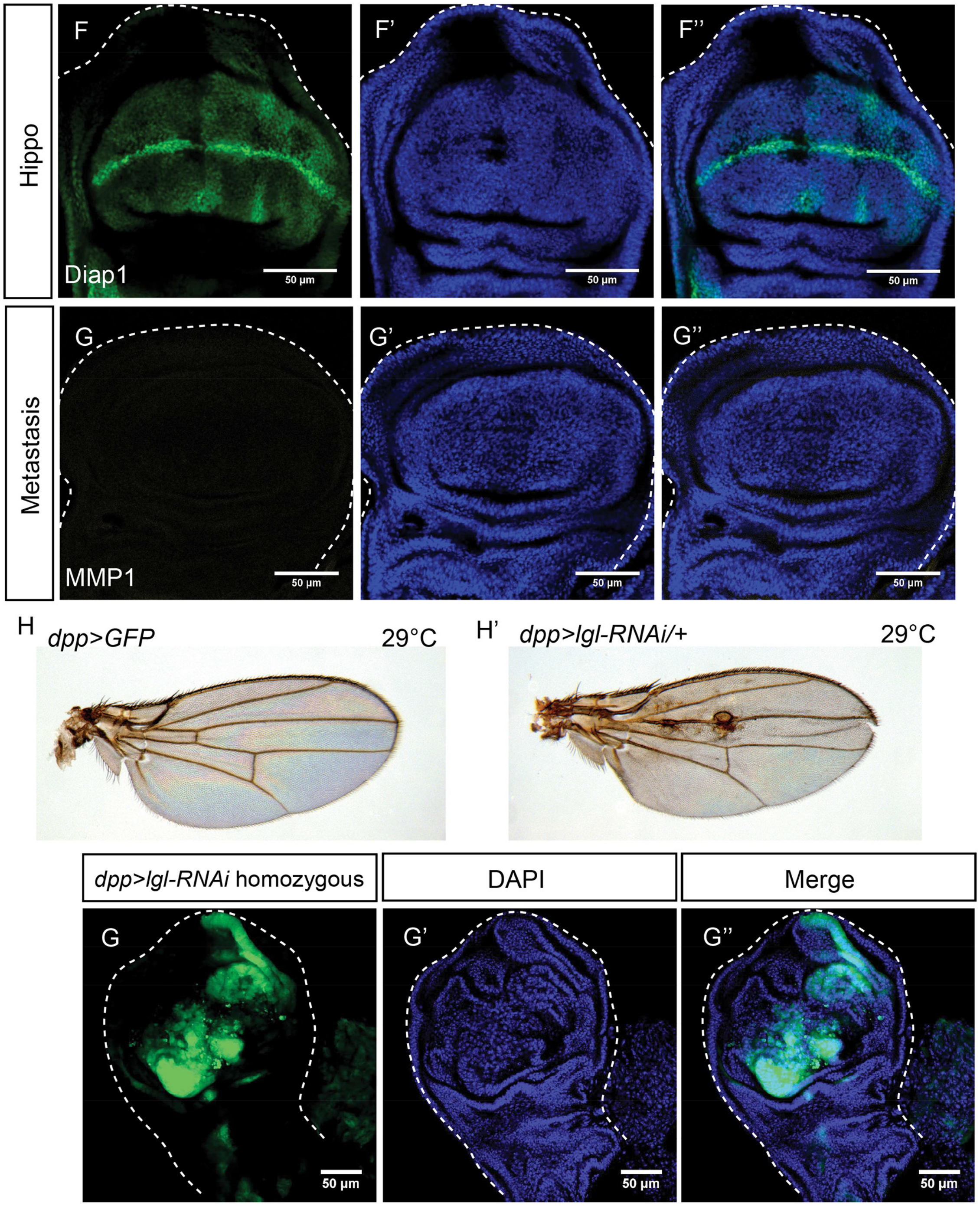

Supplementary Figure 5: The phenotypes of *dpp>lgl-RNAi* **(A-C")** Several signaling pathways and biological processes, including insulin/insulin-like signaling (A-A"), JNK signaling (B-B"), and apoptosis (C-C"), were affected in *dpp>lgl-RNAi*. The signals were reported by tGPH, TRE, and Dcp1, respectively. The arrows indicate upregulation of signals **(D-G")** Notch (D-D"), JAK/STAT (E-E"), and Hippo (F-F") signaling pathways were not obviously affected in *dpp>lgl-RNAi*. Metastasis marker MMP1 was not detected in this genotype (G-G"). The signals were reported by NRE, STAT, Diap1, and MMP1, respectively. Scale=50um. **(H-H')** Defects in the adult wings of heterozygous *dpp>lgl-RNAi* (H'), compared with the normal morphology in *dpp>GFP* (H) at 29 °C. (G-G"). Homozygous *dpp>lgl-RNAi* in the wing disc showed the tumorigenic phenotype. Scale=50um.

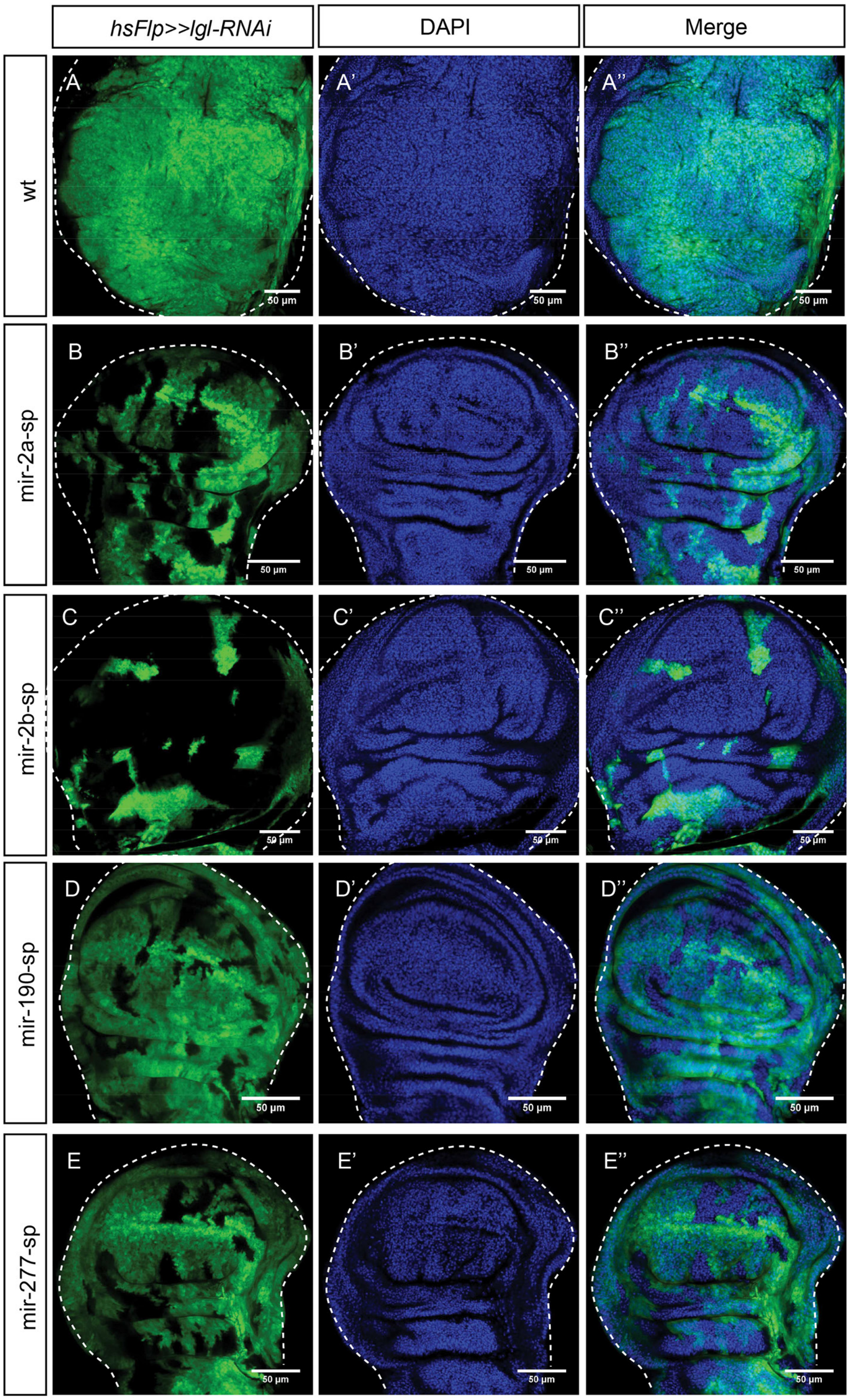

Supplementary Figure 6: Inhibition of tumor-enhancing miRNAs represses lgl defect-induced tumorigenesis **(A-A")** *lgl* defect induced by *lgl-RNAi*, driven by *hsFlp>>GFP*, in the wing disc. **(B-B")** The sponge lines of the tumor-suppressing miRNAs mir-2a (B-B'"'), mir-2b **(C-C")**, mir-190 **(D-D")**, and mir-277 **(E-E")** were individually expressed in the *hsFlp>>lgl-RNAi* background. The GFP signal shows gene overexpression. Scale=50um.

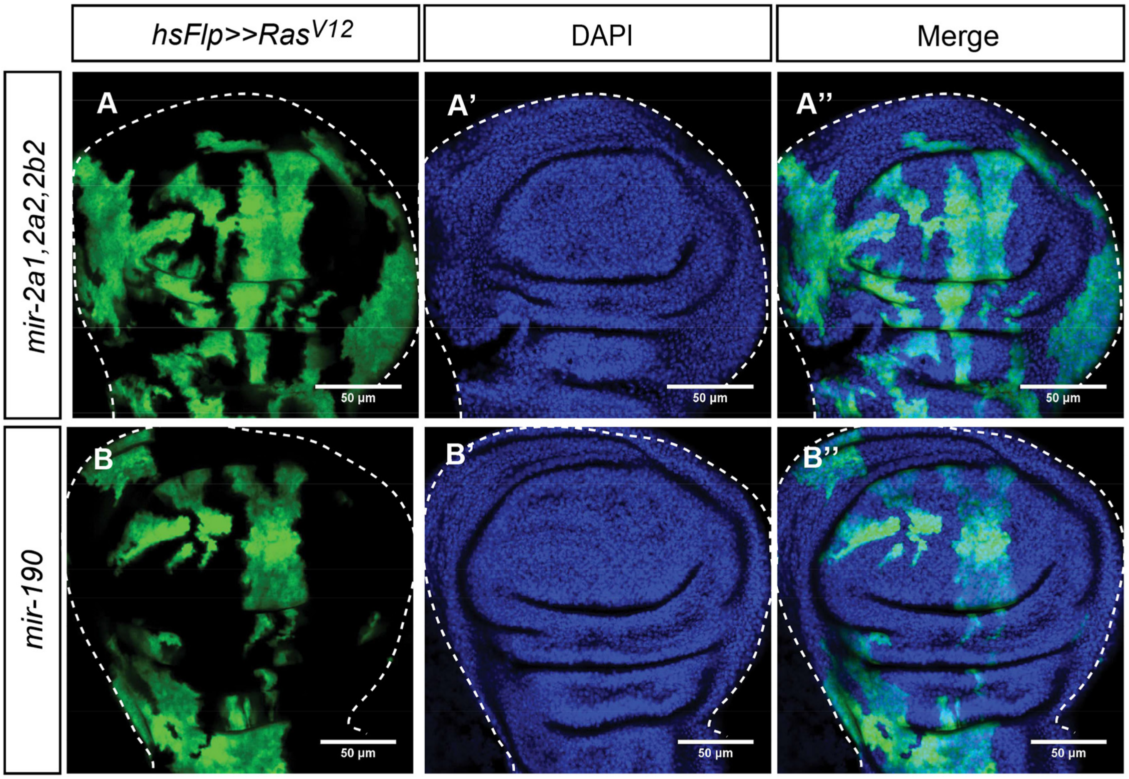

Supplementary Figure 7: Coexpression of tumor-enhancing miRNAs and *Ras*^{V12} does not induce tumorigenesis **(A-B")** mir-2a1,2a2,2b2 (A-A") and mir-190 (B-B") were coexpressed with Ras, respectively, by *hsFlp>>GFP***.** The GFP signal shows gene overexpression. Scale=50um.

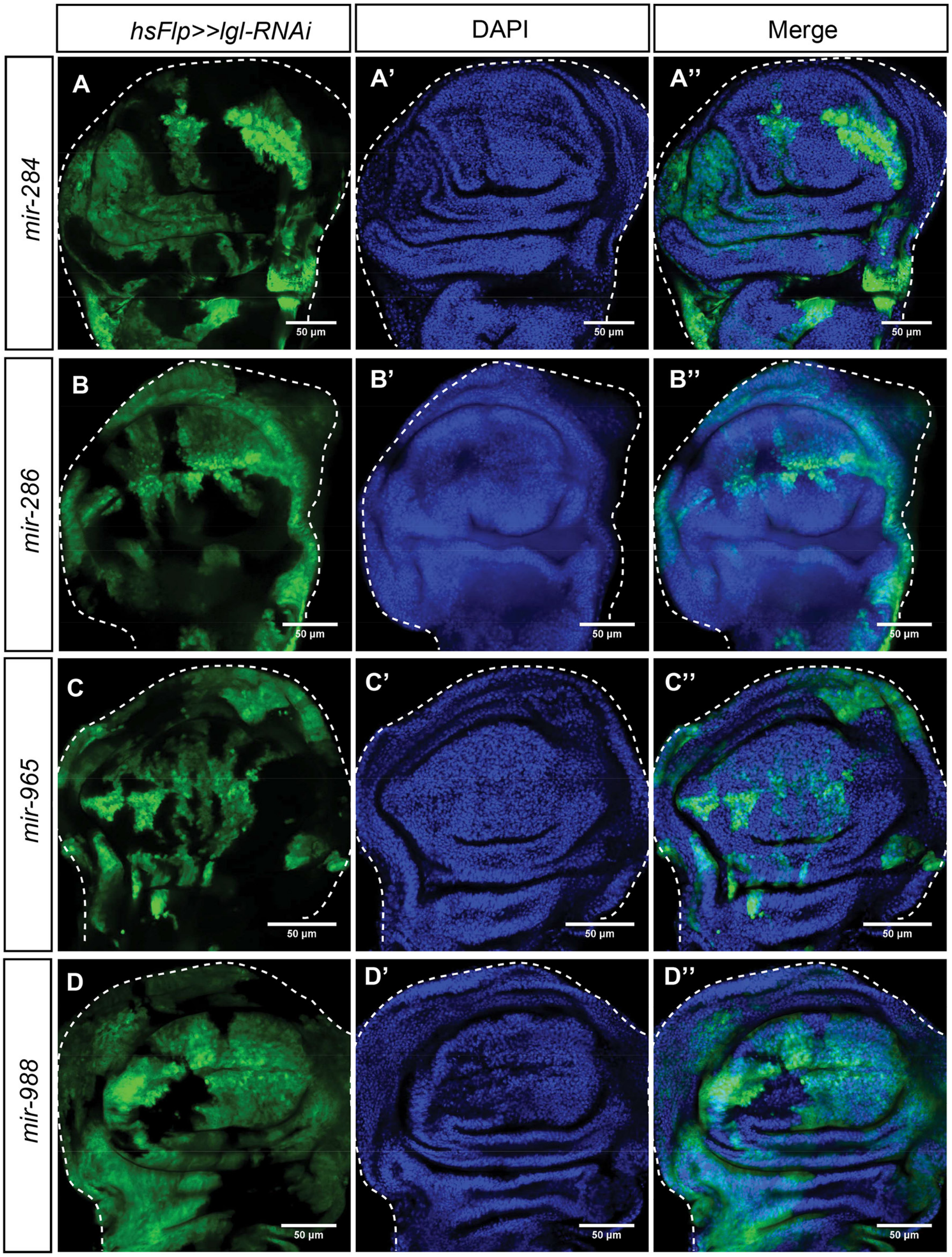

Supplementary Figure 8: Additional tumor-suppressing miRNA phenotypes **(A-D")** Tumor-suppressing miRNAs, including mir-284 (A-A"), mir-286 (B-B"), mir-965 (C-C"), and mir-988 (D-D"), were individually expressed in the *hsFlp>>lgl-RNAi* background, and suppressed tumorigenesis. The GFP signal shows gene overexpression. Scale=50um.

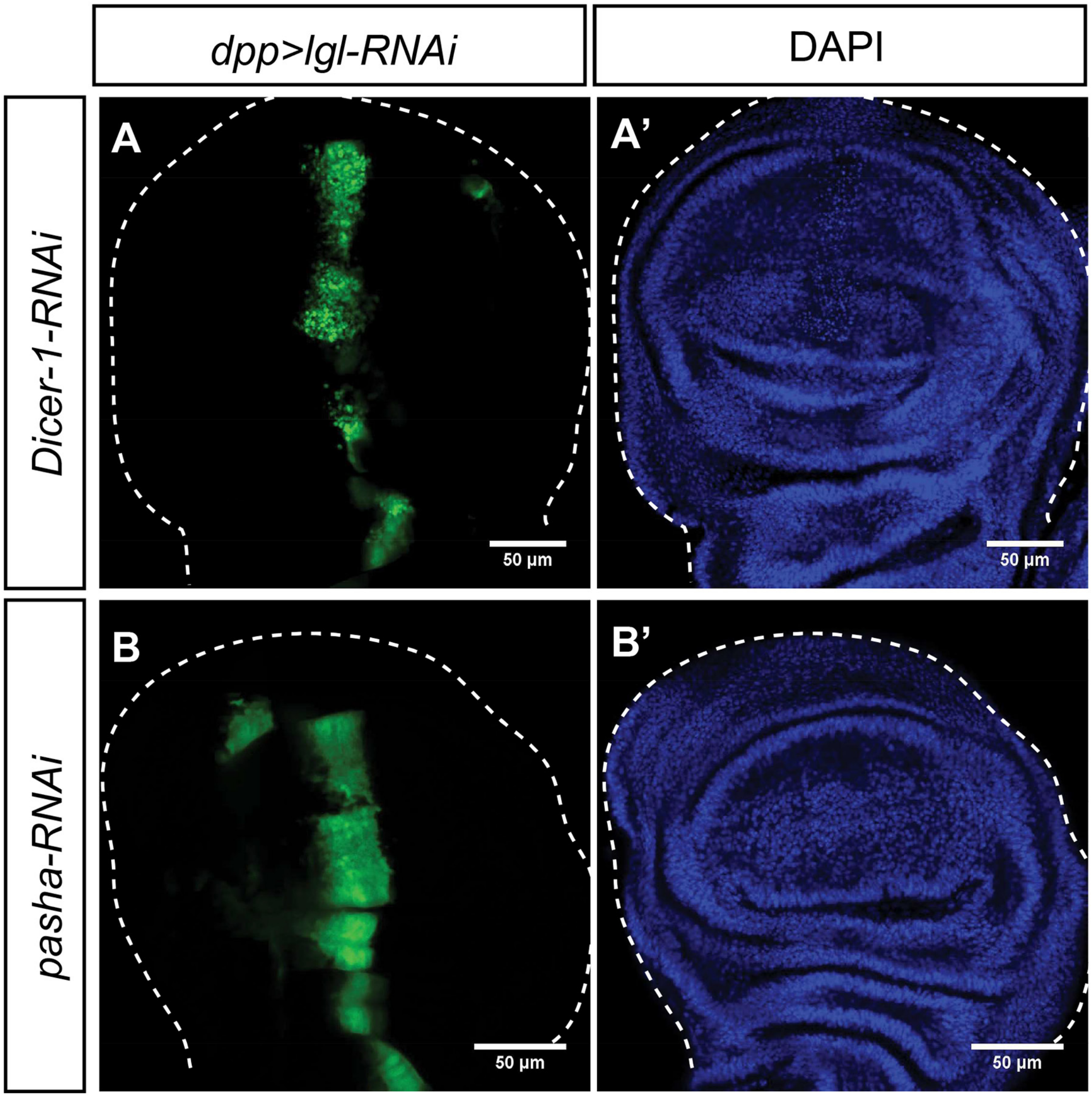

Supplementary Figure 9: Disrupting miRNA biogenesis does not enhance tumorigenesis (A-B'). Disruption of the miRNA biogenesis pathway by knocking down Dicer-1 (A-A') or pasha (B-B') did not collaborate with *dpp>lgl-RNAi* to induce tumorigenesis.

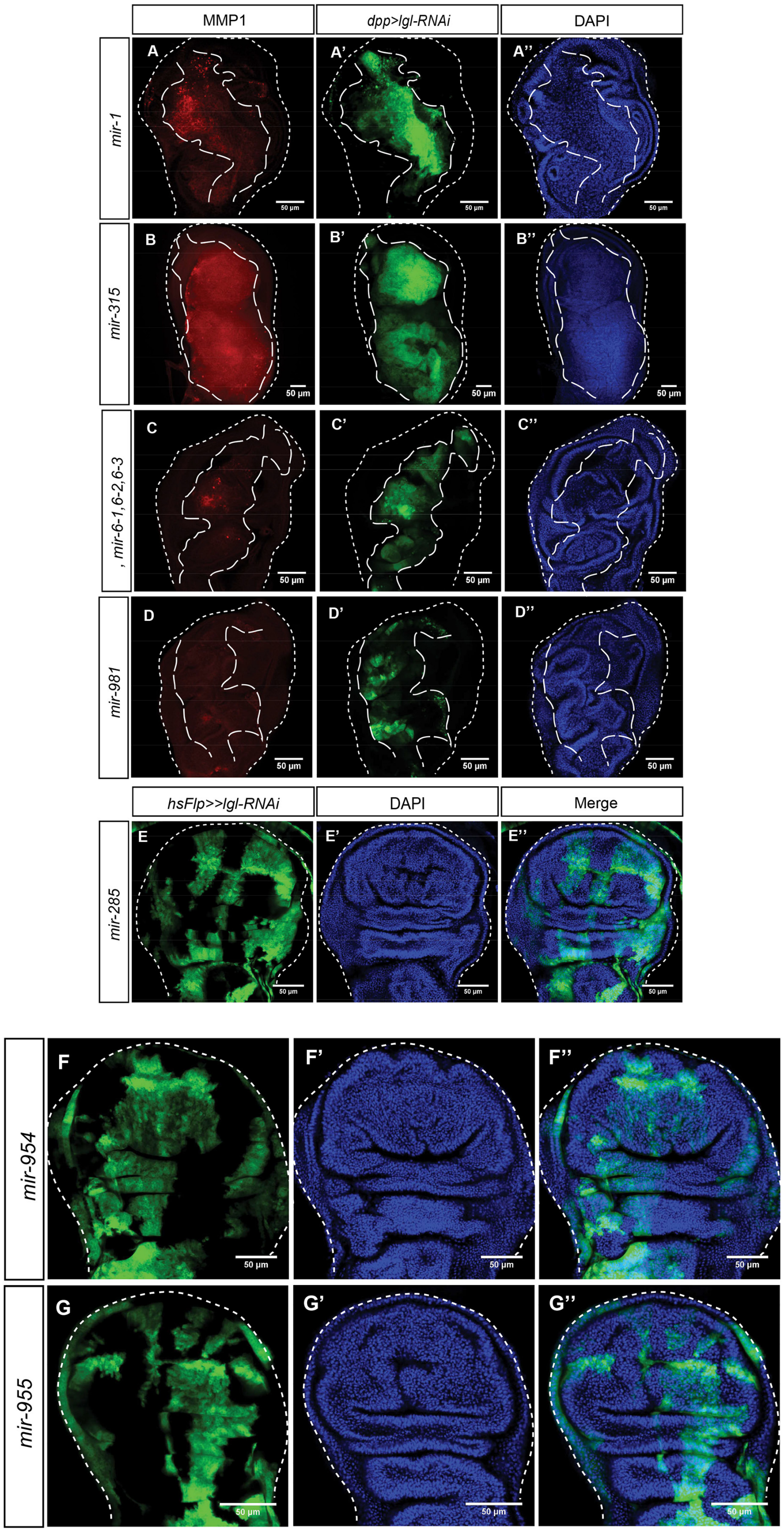

Supplementary Figure 10: Tissue specificity and miRNAs (A-D"). mir-1 (A-A"), mir-315 (B-B"), mir-6-1,6-2,6-3 (C-C"), and mir-981 (D-D") were expressed in the *dpp>lgl-RNAi* background individually. The GFP signal shows gene overexpression and the RFP shows metastasis marker MMP1. **(E-G")** mir-285 (E-E"), mir-954 (F-F"), and mir-955 (G-G") were expressed in the *hsFlp>>lgl-RNAi* background individually. The GFP signal shows gene overexpression. Scale=50um.

Supplementary Table 1: The RNA-seq results in wildtype and tumorous wing imaginal discs Three wildtype and tumor disc samples were tested. The raw sequences were mapped to 466 mature miRNAs (Materials and Methods for more details). In cases where no reads were detected in all three samples, an average of 0.33 read was used for log calculation purposes, as recorded in the Modification columns. The miRNA change in epithelial tumors was calculated as the log2 value of the read percentage in tumors / the read percentage in the wildtype

See Supplementary File 1

Supplementary Table 2: Fly stocks used in functional genetics A total of 23 and 20 fly stocks were acquired to test tumor-enhancing and tumor suppressing effects of upregulated and downregulated miRNAs, respectively

See Supplementary File 2

Supplementary Table 3: miRNA target gene analysis miRNAs that can bind to *lgl* mRNA or *Ras* mRNA are listed in the first tabs

See Supplementary File 3

Supplementary Table 4: *Drosophila* miRNA conservation in humans 26 precursor miRNAs (52 mature miRNAs) in *Drosophila* are conserved in humans, based on the gene family analysis on miRbase. Conserved miRNAs are highlighted in yellow. Tumor-enhancing and -suppressing miRNAs identified in this study is marked 892 with green and red, respectively

See Supplementary File 4

Supplementary Table 5: Primers used in qRT-PCR Primers were designed based on the sequence of each miRNA and prepared for qRT-PCR

See Supplementary File 5
